# Dendritic Spine Plasticity and Cognition

**DOI:** 10.1155/2012/875156

**Published:** 2012-05-28

**Authors:** Ignacio González Burgos, Irina Nikonenko, Volker Korz

**Affiliations:** ^1^Laboratorio de Psicobiología, División de Neurociencias, CIBO, IMSS, Guadalajara, JAL 44340, Mexico; ^2^Departamento de Biología Celular y Molecular, CUCBA, Universidad de Guadalajara, Guadalajara, JAL 45110, Mexico; ^3^Department of Neuroscience, Geneva University School of Medicine, 1211 Geneva, Switzerland; ^4^Otto von Guericke University Magdeburg, Institute of Biology, Leipziger Straße 44, Building. 91, 39120 Magdeburg, Germany

The scientific studies of dendritic spines have experienced an overwhelming growth in the last three decades. Since the primary concept that dendritic spines are sites of excitatory synaptic contact, the most recent studies have led to theoretical proposals that relate spines' morphophysiology with the information processing that underlies cognition. Furthermore, some specific spine shapes have been related to specific components of cognitive process, such as acquisition of information (learning) or long-term information retention (memory).

Dendritic spines are highly dynamic structures. In fact, their structural modifications occurring under specific conditions are strongly related to synaptic plasticity which underlies cognitive flexibility. This provides the ability to create a mnemonic trace that defines the individual experience and allows making useful decisions under different circumstances.

Contributions to this special issue provide some theoretical and experimental evidence related to the dendritic spine and synapse plasticity underlying mnemonic activity, as well as modulatory mechanisms involved in it. Although the data discussed in this special issue deal mainly with the findings derived from the animal research, it is clear, however, that these experimental results can lead to the clinical approaches concerning a wide variety of neuro-psychopathological conditions in humans.

The mechanisms that regulate spine and synapse morphology and function are characterized by the remarkable complexity. An example of such complex mechanism is described in the study by Chapleau and Pozzo-Miller that reveals the opposing effects of p75NTR and Trk receptors signaling in BDNF actions on spine density and morphology. The functional interplay between neuronal activity and different signaling pathways can induce new spine growth and affect spine maturation and stabilization/pruning, the processes that are essential for learning and memory. Another pathway regulating dendritic spine structural plasticity was studied by González-Burgos et al. They provided the evidence that estrogen receptor modulators raloxifene and tamoxifen can rapidly modify spine density and morphology and discussed the possible repercussion of these spine plastic changes on learning and memory processes. These data, obtained in experimental animals, are also of interest for the practical medicine that uses both drugs in the human treatment, and they once again highlight the importance of the fundamental knowledge for the clinical applications. 

Based on the fact that causal relationships between cellular changes underlying synaptic plasticity and their functional significance are difficult to establish from experimental studies in the mammalian brain, Giachello et al. review some of the actual evidence regarding the cellular and molecular mechanisms underlying synaptogenesis and synaptic plasticity in the invertebrate nervous system and their possible validation in vertebrates. According to this view, Lee et al. discuss some of the actual trends in the conceptualization of dendritic spines as discrete functional compartments regulating synaptic plasticity, as well as the spine's structural changes associated with synaptic function.

In other review papers presented in this issue, the mechanisms regulating normal brain plasticity and their implications in the pathological processes are discussed. Mandela and Ma focus on Kalirin-7, a Rho guanine nucleotide exchange factor that plays important role in signaling pathways regulating formation of dendritic spines and synapses. Recent data demonstrate that Kalirin-7 not only affects spine and synapse morphology but, consequently, modulates plasticity properties of a synapse. More importantly, human analogue of this rodent protein is implicated in a wide range of human diseases related to the cognitive disability. The paper by Bitzer-Quintero and Gonzalez-Burgos reviews the role of the immune system in regulation of synaptogenesis, especially under conditions of brain injury or inflammation. Increasing data also suggest implication of the complex interactions between immune and nervous systems in the normal neuronal structural plasticity underlying learning and memory mechanisms.

We hope that papers published in this special issue will serve to increase the scientific knowledge on the cellular and molecular mechanisms involved in dendritic spine's plastic changes underlying cerebral organization of learning and memory.

*Ignacio González Burgos* *Ignacio González Burgos* *Irina Nikonenko* *Irina Nikonenko* *Volker Korz* *Volker Korz*

